# Roselle (*Hibiscus sabdariffa* L.) extract as an adjunct to valsartan in patients with mild chronic kidney disease: A double-blind randomized controlled clinical trial

**DOI:** 10.22038/AJP.2024.23871

**Published:** 2024

**Authors:** Behdad Dehkhoda, Ayesheh Enayati, Hassan Mirzaei, Somayeh Ghorbani, Mohammad Hadi Soleimani, Saeid Amirkhanlou, Amirhossein Sahebkar

**Affiliations:** 1 *Ischemic Disorders Research Center, Golestan University of Medical Sciences, Gorgan, Iran*; 2 *Rheumatology Research Center, Golestan University of Medical Sciences, Gorgan, Iran*; 3 *Giah Essence Phytopharm-Dr. Soleimani Co.*; 4 *Biotechnology Research Center, Pharmaceutical Technology Institute, Mashhad University of Medical Sciences, Mashhad, Iran *; 5 *Applied Biomedical Research Center, Mashhad University of Medical Sciences, Mashhad, Iran*; † *Equal first author*

**Keywords:** Chronic kidney disease, Hypertenstion, Valsartan, Hibiscus sabdariffa, Antocyanidin, Hydrochlorothiazide, Lipid profile, Molecular docking

## Abstract

**Objective:**

The objective of this study was to evaluate the effectiveness of *Hibiscus sabdariffa* L. extract (HS) as an adjunct to valsartan in the treatment of high blood pressure in patients with mild chronic kidney disease (CKD).

**Materials and Methods:**

This trial was conducted in Gorgan, Iran. Seventy-two participants with CKD and high blood pressure were randomly assigned to either the HS group, receiving a 350 mg pill every 12 hr for 90 days along with 40 mg of valsartan every 12 hr, or the control group (40 mg valsartan + 12.5 mg hydrochlorothiazide). The primary objective was to assess the improvement of hypertension, while secondary objectives included the evaluation of proteinuria, albuminuria, kidney function, lipid profile, and electrolyte levels. Molecular docking analysis was performed to examine the mechanisms of action of the isolated components of HS.

**Results:**

Out of 80 initial participants, 72 were included in the analysis. Both groups showed a significant reduction in blood pressure (p<0.001). The HS group demonstrated a statistically significant decrease in lipid profile (p<0.001). There were no statistically significant differences between the groups regarding the reduction of renal markers. Molecular docking analysis revealed that the compounds present in HS, particularly its anthocyanins and flavonoids, exhibited greater angiotensin-converting enzyme (ACE) inhibitory potential than hydrochlorothiazide in both domains. Moreover, the compounds met the criteria for drug likeness and Lipinski rules.

**Conclusion:**

Adjunctive therapy with HS showed promising results in reducing hypertension and improving lipid profile in patients with CKD.

## Introduction

Chronic kidney disease (CKD) is a rapidly growing non-communicable disease as well as a serious global public health concern (Liyanage et al., 2022 ▶; Cockwell and Fisher, 2020 ▶). Clinically, CKD is defined by a glomerular filtration rate (GFR) below 60 ml/min/1.73 m² and albuminuria as markers of kidney damage (Liyanage et al., 2022 ▶). Diabetes, hypertension, and cardiovascular disease are major contributors to CKD. However, cardiovascular diseases are known as leading cause of premature morbidity and mortality in CKD patients (Liyanage et al., 2022 ▶; Cockwell and Fisher, 2020 ▶).

CKD and hypertension are interconnected risk factors, as hypertension promotes the progression of CKD, and declining estimated glomerular filtration rate (eGFR) increases the incidence and severity of hypertension (Hamrahian and Falkner, 2017 ▶; Pugh et al., 2019 ▶). Pharmacological approaches for managing hypertension in CKD patients include angiotensin-converting enzyme inhibitors, angiotensin II receptor blockers (ARBs), and diuretics, either prescribed individually or in combination based on eGFR (Hamrahian and Falkner, 2017 ▶; Pugh et al., 2019 ▶). However, the complex interaction between hypertension and CKD, especially when these conditions coexist, contributes to an increased risk of cardiovascular disease and adverse cerebrovascular outcomes (Pugh et al., 2019 ▶). Given the rising prevalence of hypertension in CKD patients (Muntner et al., 2010 ▶), adopting an adjunctive approach is essential to improve hypertension management.

Herbal medicines have been widely used worldwide based on positive beliefs and experiences, for treating various diseases (Jalalyazdi et al., 2019 ▶). *Hibiscus sabdariffa* L. (hibiscus, roselle, or sour tea) from the Malvaceae family has been traditionally and scientifically recognized for its antihypertensive properties (Ellis et al., 2021 ▶; Jalalyazdi et al., 2019 ▶; Sakhaei et al., 2020 ▶). Furthermore, *Hibiscus sabdariffa* L. extract (HS) HS has shown beneficial effects in management of several chronic diseases and disorders, including cancer, diabetes, oxidative stress, cardiovascular diseases, and inflammation (Ellis et al., 2021 ▶; Sakhaei et al., 2020 ▶). HS has also been reported to possess nephro- and hepato-protective, anti-hypercholesterolemic, diuretic, and anti-obesity properties (Manzano-Pech et al., 2022 ▶; Sakhaei et al., 2020 ▶). Previous studies have attributed these biological effects to the chemical constituents present in HS, such as anthocyanins, flavonoids, polysaccharides, and organic acids (Ellis et al., 2021 ▶; Hopkins et al., 2013 ▶; Manzano-Pech et al., 2022 ▶).

Likewise, animal and randomized controlled trial (RCT) studies have demonstrated that the consumption of roselle can significantly improve blood pressure compared to captopril, which reduces both systolic and diastolic blood pressure in patients with hypertension and type 2 diabetes. In addition, HS has exhibited beneficial effects in lipidemic, hyperlipidemic, and diabetic animal models by reducing lipid profile parameters such as cholesterol, low-density lipoprotein cholesterol (LDL-C), and triglycerides (TG) without affecting high-density lipoprotein cholesterol (HDL-C). Some RCTs have reported that HS tea/extract decreases lipid profile and increases HDL-C (Hopkins et al., 2013 ▶). The rich anthocyanin content of HS, combined with these findings, can explain the hypotensive and anti-cholesterol roles of HS through its antioxidant effects. Another clinical study demonstrated that consumption of 425 mg of HS pill (hydroalcholic extract of flowers) twice daily remarkably reduced systolic blood pressure, high-sensitivity C-reactive protein, and renal factors (blood urea nitrogen and creatinine, and urine creatinine and albumin) in patients with diabetic nephropathy (Sakhaei et al., 2021 ▶). Moreover, administration of HS tea twice a day for one month significantly decreased blood pressure in participants with stage one hypertension and modified lifestyle and dietary (Jalalyazdi et al., 2019 ▶).

Due to the regular consumption of HS as part of a balanced nutrition and considering its safety and tolerance (Ellis et al., 2021 ▶; Hopkins et al., 2013 ▶), we designed a double-blind, randomized controlled study to investigate the effect of HS pills compared with hydrochlorothiazide on improving hypertension and renal factors in patients with hypertensive nephropathy. Additionally, we aimed to evaluate the molecular docking of some isolated components of HS to identify their mechanisms of action.

## Materials and Methods

**Study design**

This was a double-blind randomized controlled trial conducted on CKD patients from September 2021 to March 2022, at a single center; patients were admitted to the Sayyad Medical and Eductional Center, Golestan University of Medical Sciences, in the north of Iran.

**Study population**

Highly probable patients of CKD (stage I or II) between 18 and 70 years of age having hypertension with systolic blood pressure (SBP) ≥ 130 mmHg and diastolic blood pressure (DBP) ≥ 80 mmHg (Whelton et al., 2017 ▶) for outpatient treatment entered the trial. Exclusion criteria were having SBP≤ 120 mmHg, DBP≤ 80 mmHg, diabetes mellitus, severe renal failure (stage III or V with GFR <60 ml/min/1.73m²), coronary artery disease, heart failure, metabolic disease, mental illness, pregnancy or lactation, use of other herbal medicines to control the symptoms of the disease, sensitivity to HS, or need to be hospitalized.

**Ethics**

The study had been approved by the Ethics Committee of Golestan University of Medical Sciences (IR.GOUMS.REC.1400.229) and registered at the Iranian Registry of Clinical Trials (IRCT20210926052600N1). All patients were informed and they signed the written informed consent to participate in the trial. 

**Roselle pill preparation and formulation**

The samples of HS aerial parts were obtained from a local medicinal store in Mashhad, Iran. The plant was authenticated and deposited at the Herbarium of the Faculty of Pharmacy, Tehran University of Medical Sciences, with the herbarium number (PMP-2381).

To prepare the pill, 150 g of aerial parts of roselle was washed and ground. The powdered material was then subjected to extraction using aqueous ethanol (70%) through the percolation method. The solvent from the extract was removed using a rotary evaporator under vacuum at 40°C. The concentrated extract obtained was subsequently dried and converted into powder form using spray drying. The pill was prepared by incorporating 350 mg of HS extract powder (standardized based on anthocyanin content) into a 500 mg pill, following standard guidelines for pill manufacturing. All steps involved in the preparation of the drug, including extraction and pill-making, were carried out at the Giah Essence Phytopharmaceutical Dr. Soleimani Company.

**Clinical evaluation**

**Sample size**

Considering urinary albumin and blood pressure as the main outcomes, and assuming a 10% loss to follow-up, the sample size of 36 patients was calculated in each group (power=90%; α=0.05). The eligible patients were allocated into two groups using block randomization method. Interventions were specified for all possible forms of blocks in size four (different permutations of two interventions at two times) and then were randomly selected. At the end, a random list was created with the contents of the participants’ numbers and their assigned intervention. For blinding and concealment, a unique code was given to each participant and the same code was attached to their drug package; also, the care providers were blind to the allocation of patients.

**Intervention**

In the HS group (intervention), the participants received 500 mg of HS pill per 12 hr (twice a day) plus 40 mg valsartan pill per 12 hr, and the control group patients received 40 mg valsartan plus 12.5 mg hydrochlorothiazide per 12 hr, for 90 days. The patients were followed up during the study through a face-to-face visit and telephone contact.

**Measurements**

The primary measurement was reduction in hypertension and urinary albumin of CKD patients after 90 days of intervention. The interval of endpoint measurement against the baseline was considered as the decrease in blood pressure. Blood pressure was taken according to the guidelines by the Riester Nova Mercury barometer and the fixed-expert nurse who was unaware of the study. The secondary measurements were microalbuminuria, proteinuria, eGFR, blood urea nitrogen (BUN), serum electrolytes, creatinine, and lipid profile. The urinary albumin and the secondary measurement were evaluated in baseline and after the 90 days of intervention. 

**Molecular docking**

In this study, the plausible selective-angiotensin-converting enzyme (ACE) inhibition (cACE or nACE domains) potential of HS compounds (23 compounds, Table 3) was investigated by *in silico* docking software Auto Dock (4.2) on nACE-inhibition (PDB ID: 6F9V) and cACE-inhibition (PDB ID: 6F9U) complexes (Caballero, 2020; Xu et al.,2022). Also, effects of the compounds on the kidney target proteins such as Alpha-1-microglobulin (PDB ID: 1BIK), Beta-2-microglobulin (PDB ID: 2XYF), Alpha-1-acid glycoprotein 1 (PDB ID: 3QKG), Complement factor D (PDB ID: 1DIC), Prostaglandin-H2 D-isomerase (β-Trace protein) (PDB ID: 2WWP), Retinol-binding protein 4 (PDB ID: 3FMZ), and Cystatin-C (PDB ID: 3GAX) were assessed by molecular docking. All performed docking was compared with hydrochlorothiazide as a control. The water molecules/non-polar hydrogen atoms of the target proteins were removed and then target proteins were prepared by adding polar hydrogens/kollman charges for docking study (Xu et al., 2022 ▶). Lamarckian genetic algorithm (GA) was used and 150 GA runs were accomplished for each docking. The visualization of 2D and 3D presentation was revealed by Maestro 11.0 Schrodinger suit and Discovery Studio Visualizer software. Additionally, the 3D structure of HS compounds (as ligands) obtained from PubChem database and then Open Babel software was used to convert their structures into PDB format. Chem3D software was utilized for energy minimization of ligands. 

**Drug likeness and ADMET prediction**

AdmetSAR database and SwissADME were applied to predict efficacy and safety of the HS compounds and their pharmacokinetics through drug likeness and absorption, distribution, metabolism, excretion and toxicity (ADMET) prediction. Likewise, we evaluated effects of the mentioned compounds on topological polar surface area (TPSA), blood–brain barrier (BBB) permeability, cytochrome P450 (CYP450) inhibition, assess the mutagenic potential of chemical compounds (AMES) toxicity and carcinogenicity (Xu et al., 2022 ▶).

**Statistical methods**

Data are presented as median with interquartile range for non-normally distributed variables and as means±SEM for normally distributed continuous variables. Normally distributed variables were analyzed using the student’s t-test, whereas non-normally distributed variables were analyzed using the Mann–Whitney’s U-test to compare demographic and laboratory data between the HS and control groups. All p values <0.05 were considered statistically significant. Statistical analyses were conducted using SPSS version 22.0 (IMB SPSS Inc., New York, United States).

## Results

**Baseline characteristics**

**Figure 1:**
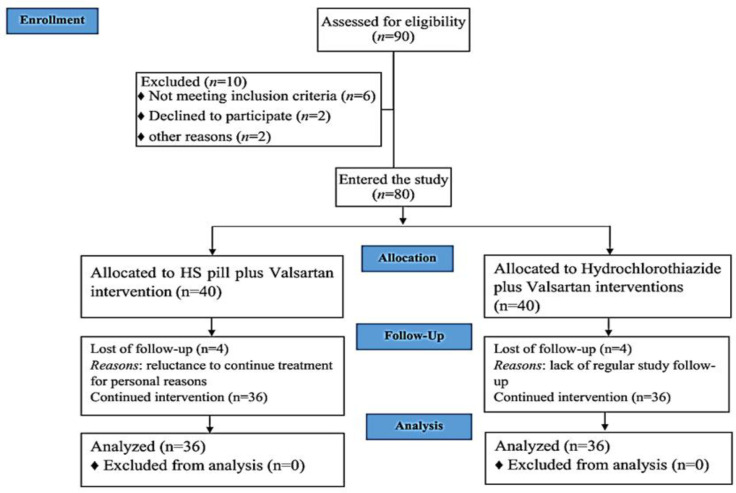
CONSORT flowchart of the study

According to the CONSORT flowchart (Figure 1), a total of 90 participants were assessed for eligibility between September 2021 and March 2022. Ten individuals were excluded from the study, with six not meeting the inclusion criteria, two withdrawing the consent, and two for other reasons. Ultimately, 80 participants were enrolled in the study, with 40 receiving HS pill plus valsartan interventions and 40 receiving hydrochlorothiazide plus valsartan interventions (control group). During the course of the study, four participants in the HS group discontinued the HS pill due to personal reasons, and four participants in the control group did not have regular study follow-up. Ultimately, 72 patients (36 in the HS group and 36 in the control group) completed the study and were included in the analysis.

The average age of the participants was 53.32±10.76 years, with a distribution of 39 (54.2%) males and 33 (45.8%) females. The participants ranged in age from 18 to 70 years, with 19 (52.8%) males and 17 (47.2%) females in the HS pill group, and 20 (55.6%) males and 16 (44.4%) females in the control group (Table 1). All participants were from Gorgan, Golestan, Iran. At baseline, there were no statistically significant differences in demographic and clinical characteristics between the groups.

**Table 1:** General characteristics of the patients

	Control group	HS group	p-value
Age (years) (mean ± SD)	53.22 ± 10.03	52.64 ± 11.30	0.813
Sex N (%)	20 (55.6) Male 16 (44.4) Female	19 (52.8) Male 17 (47.2) Female	0.998

SD=standard deviation

**Efficacy of the treatment**

**Primary outcomes**

Statistically significant differences were observed in the primary endpoint, blood pressure, between baseline and end of intervention in the two groups (p<0.001) (Table 2). Also, the baseline SBP was reduced from 145 mmHg to 130 (control group) or 140 (HS group) mmHg by the end of study. The control group showed a reduction in urinary albumin levels, although the difference was not statistically significant compared to the HS group. At the 3-month mark, changes in SBP and DBP were significantly higher in the control group than in the HS group (p=0.001 and p=0.009, respectively) (Table 2). Additionally, urinary albumin levels decreased in the control group compared to baseline within the 90-day intervention period (p<0.05).

**Table 2:** The variables of CKD patients in both groups

**Variables**	**Group**	**N**	**Baseline**	**After 3 months intervention **	p value*	**Changes** **	**p value** ***
**SBP (mmHg)**	1	36	145 [140, 160]	130 [120, 140]	< 0.001	17.5 [15, 23.8]	0.001
	2	36	145 [140, 163.8]	140 [130, 150]	< 0.001	10 [10, 15]	
**DBP (mmHg)**	1	36	95 [90, 100]	80 [76.3, 83.8]	< 0.001	15 [10, 18.8]	0.009
	2	36	90 [85, 95]	80 [80, 90]	< 0.001	10 [5, 15]	
**GFR (ml/min/1.73m²)**	1	36	67.35 [63.1, 73.9]	68.05 [56.3, 78.8]	0.607	3.65 [-7.7, 9.4]	0.906
	2	36	66.40 [63.4, 76.9]	69.75 [58.6, 78.4]	0.879	5 [-9.3, 9.2]	
**Upr24hr (mg)**	1	36	150 [93.2, 220]	117.5 [77.3, 160.3]	0.109	10.50 [-25.8, 78.8]	0.919
	2	36	137.50 [102.3, 307.5]	142.50 [101.5, 235]	0.311	-2 [-25, 53.8]	
**Ualb24hr (mg)**	1	36	34 [20.9, 56]	23.5 [15.1, 31]	0.017	5 [-1.8, 20.5]	0.386
	2	36	26.70 [20.5, 61.5]	28.50 [20.2, 47]	0.460	-0.35 [-5.2, 18.6]	
**Bun (mg/dL)**	1	36	21 [18, 26.6]	22 [17.5, 32.5]	0.106	-1.5 [-6.2, 2.8]	0.543
	2	36	18 [15, 27.87]	21 [17, 32]	0.042	-2.5 [-12.6, 3]	
**Cr(mg/dL)**	1	36	0.98 [0.9, 1.1]	1 [0.9, 1.2]	0.244	-0.04 [-0.1, 0.09]	0.690
	2	36	1 [0.9, 1.1]	1 [0.9, 1.2]	0.197	-0.1 [-0.1, 0.1]	
**Na** **(mEq/L)**	1	36	140 [138.1, 143.8]	139 [138.1, 140.1]	0.184	1.1 ± 4.7	0.161
	2	36	139.4 [137, 142.8]	140 [138, 142.8]	0.231	-0.7 ± 5.7	
**K (mEq/L)**	1	36	4.05 [3.7, 4.3]	4.7 [4, 4.8]	0.182	-0.2 [-0.38, -0.2]	0.113
	2	36	4.1 [4, 4.6]	4.5 [4, 4.8]	0.385	0.1 [-0.3, 0.6]	
**Cholesterol (mg/dL)**	1	36	170 [151.3,191.5]	173 [145.3, 196.8]	0.540	2.5 [-7.8, 12.5]	0.002
	2	36	162 [145, 186.7]	147.50 [130, 168]	< 0.001	17 [5.3, 24.8]	
**TG (mg/dL)**	1	36	179 [152, 220]	178 [153, 210.3]	0.919	-10 [-21.3, 27]	0.011
	2	36	163.5 [140.3, 186.8]	144 [123, 163]	< 0.001	13 [7.3, 26.3]	
**LDL-C (mg/dL)**	1	36	85 [78, 99.5]	88 [77, 109.5]	0.322	-2.4 [-12.8, 7]	0.104
	2	36	89 [75.5, 98.3]	82 [75, 95]	0.266	6.5 [-13.8, 22.8]	
**HDL(mg/dL)**	1	36	45 [37.9, 52.8]	40.45 [36, 49]	0.139	3.5 ± 13.8	0.823
	2	36	45.5 [41, 54]	43 [37, 49]	0.105	4.3 ± 13.7	

Group 1= control and group 2 =HS; *: Mann-Whitney U test; **: Changes from baseline to month 3; ***: T-test or Mann-Whitney U test. CKD: chronic kidney disease; SBP: Systolic blood pressure; DBP: diastolic blood pressure; GFR: glomerular filtration rate; HDL-C: high-density lipoprotein cholesterol; TG: triglycerides; LDL-C: low-density lipoprotein cholesterol; BUN: Blood urea nitrogen; Cr: Creatinine; K: Potassium; Na: sodium; Upr24hr= 24 hr urine protein; UAlb24hr: 24 hr urinary albumin; SD=standard deviation; BMI: Body mass index. Median [interquartile range] for non-normally distributed variables, and means ± standard deviations for normally distributed continuous variables.

**Secondary outcomes**

After 3 months, the HS group exhibited reductions in cholesterol and TG levels, with significant differences observed between the groups (p=0.002 and p=0.011, respectively) (Table 2). Furthermore, there were statistically significant differences in both cholesterol (from 162 to 147.5 (mg/dL)) and TG (from 163.5 to 144 (mg/dL)) levels at the end of the observation period compared to baseline (p<0.001). In addition, HS decreased LDL-C level with no statistically significant differences between the baseline and end of study. While, thiazide slightly with no statistically significant increased all lipid indexes. However, reduction of lipid profile by HS showed that it doesn’t has side effects of thiazide but there are needed larger RCTs. Other secondary endpoints did not show significant changes in either group compared to baseline by the end of the observation period (Table 2).

**Safety of the treatment**

No complications or adverse events related to HS pill consumption were reported during the study. Laboratory data analysis indicated no significant harmful changes in lipid profile, BUN, serum creatinine, Na, or K levels within the 90-day consumption period.

**Molecular docking**

Based on the findings presented in Table 3, the compounds found in HS demonstrated a greater selective ACE inhibitory potential compared to hydrochlorothiazide in both domains. Specifically, the anthocyanins present in HS exhibited higher selectivity in inhibiting the cACE and nACE domains, as indicated by their lower binding energies on targets 6F9U and 6F9V. Among these compounds, tiliroside displayed the lowest binding energy.

**Figure 2:**
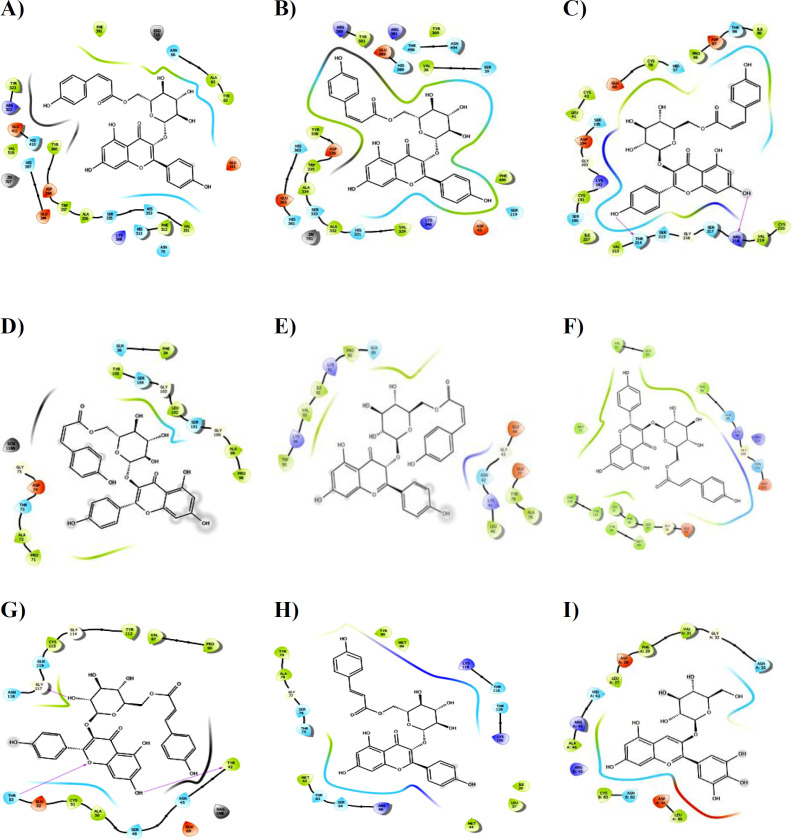
Presentation of 2D model of interactions between Tiliroside and **A)** PDB ID: 6F9U, **B)** PDB ID: 6F9V, C) PDB ID: 1DIC, D) PDB ID: 2WWP, E) PDB ID: 2XYF, F) PDB ID: 3FMZ, G) PDB ID: 1BIK, H) PDB ID: 3QKG; I) Presentation of 2D model of interactions between Delphinidin-3-glucoside and PDB ID: 3GAX

Furthermore, tiliroside interacted with the cACE receptor target (PDB ID: 6F9U) through hydrophobic, polar, and hydrogen bonds with specific amino acid residues in the active site, as shown in Table 4. A total of seven hydrogen bonds were formed, with bond distances ranging from 1.70 to 2.64 Å (Figure 2A). Similarly, tiliroside selectively inhibited the nACE receptor target (PDB ID: 6F9V) at distances ranging from 1.99 to 2.63 Å. The hydroxyl group in tiliroside played a significant role in enhancing the interaction with the binding pocket of the target and specific amino acids (Table 4, Figure 2B).

Additionally, the results indicated that HS compounds, particularly tiliroside and other anthocyanidins, acted as selective nACE inhibitors with more favorable binding energies in inflammatory-inhibitor complexes 3GAX, 3FMZ, 2XYF, 2WWP, and 1DIC, which served as kidney benchmarks, compared to hydrochlorothiazide. Notably, tiliroside exhibited the highest inhibitory effect on the 3FMZ, 2XYF, 2WWP, and 1DIC complexes in terms of binding energy and the amino acids involved (Table 3 and 4). Figure 2 illustrates tiliroside forming eight hydrogen bonds with the 1DIC target, with bond distances ranging from 2.46 to 3.38 Å (Figure 2C). In the Prostaglandin-H2 D-isomerase receptor (PDB ID: 2WWP), tiliroside interacted through ten hydrogen bonds with bond lengths of 1.70, 2.48, 1.88, 2.57, 2.99, 2.17, 1.82, 2.27, 3.22, and 3.10 Å in the binding pocket (Figure 2D). Furthermore, tiliroside was stabilized in the binding pocket of the Beta 2 microglobulin receptor (2XYF) by several amino acid residues through hydrophobic, polar, and five hydrogen interactions, with hydrogen bond lengths of 1.75, 2.06, 2.94, 1.79, and 1.80 Å (Figure 2E). Lastly, in the binding pocket of the 3FMZ receptor, tiliroside formed ten hydrogen bonds with bond lengths of 2.92, 3.00, 2.72, 2.55, 1.49, 2.15, 3.07, 2.36, 2.69, and 3.13 Å (Figure 2F). 

**Table 3:** Molecular docking simulation results for the HS compounds and receptors

Compounds	Binding Energy (kcal/mol) PDB ID:								
	1BIK	1DIC	2WWP	2XYF	3FMZ	3GAX	3QKG	6F9U	6F9V
Tiliroside	-8.1	-10.7	-7.3	-7.0	-10.1	-7.3	-7.3	-9.7	-10.2
Delphinidin-3-sambubioside	-7.3	-8.6	-6.7	-5.3	-7.9	-6.7	-6.4	-8.4	-8.7
Cyanidin 3-O-beta-D-sambubioside	-7.3	-8.5	-6.9	-6.3	-8.1	-7.1	-6.6	-8.9	-8.5
Delphinidin-3-glucoside	-6.5	-7.9	-6.0	-5.1	-8.5	-8.5	-6.1	-7.8	-7.6
Cyanidin 3-galactoside	-6.5	-7.2	-6.1	-5.1	-7.9	-5.0	-5.0	-7.1	-6.8
Galloyl ester	-5.7	-6.8	-6.1	-4.6	-7.7	-7.1	-5.9	-6.5	-6.5
Hibiscitrin	-6.3	-7.3	-5.4	-4.6	-7.3	-7.3	-5.4	-7.2	-6.8
Quercetin	-5.1	-6.3	-4.9	-4.2	-6.1	-6.1	-4.7	-5.2	-5.1
Luteolin	-5.1	-6.4	-5.0	-4.6	-6.3	-6.3	-4.5	-5.2	-5.4
Rutin	-6.6	-9.1	-5.7	-5.1	-8.9	-8.9	-6.1	-8.3	-8.5
Kaempferol	-5.2	-5.9	-4.7	-4.1	-6.3	-6.3	-4.6	-5.0	-5.1
Eugenol	-4.3	-4.7	-4.5	-3.2	-4.2	-4.7	-4.0	-4.0	-4.1
Protocatechuic acid	-6.3	-8.1	-6.0	-5.9	-8.3	-7.1	-6.7	-8.4	-8.8
Hydroxycitric acid	-3.1	-3.9	-3.2	-2.6	-3.6	-4.3	-3.2	-3.4	-3.4
Citric acid	-3.4	-4.2	-3.7	-3.2	-3.9	-3.9	-3.5	-3.6	-3.9
Malic acid	-3.7	-3.8	-4.0	-3.3	-3.8	-3.8	-3.6	-4.4	-3.9
Tartaric acid	-3.4	-3.2	-3.8	-2.6	-3.2	-3.2	-3.2	-3.6	-2.8
Ascorbic acid	-4.4	-4.4	-4.6	-3.5	-4.2	-4.2	-3.9	-3.9	-4.3
Coumaroylquinic acid	-5.6	-6.5	-6.0	-5.1	-6.7	-6.7	-4.9	-6.0	-7.0
Chlorogenic	-5.2	-6.5	-5.5	-5.0	-6.8	-6.8	-5.4	-6.3	-6.8
Gallic acid	-4.4	-4.4	-4.4	-3.1	-4.0	-4.7	-3.4	-3.7	-3.9
β-sitosterol	-6.1	-7.2	-5.5	-5.5	-8.3	-8.3	-6.2	-7.1	-7.2
Ergosterol	-6.0	-6.5	-5.0	-5.6	-8.0	-8.0	-5.7	-7.1	-6.7
Hydrochlorothiazide	-5.1	-5.6	-4.2	-4.3	-5.2	-4.2	-4.2	-4.9	-4.9

**Table 4:** Key amino acid residues between Tiliroside and the active sites of targets

Bonding type / Targets	6F9U	6F9V	1DIC	2WWP	2XYF	3FMZ	1BIK	3QKG
Hydrophilic	Tyr523, Phe391, Val518, Tyr360, Trp357, Ala356, Phe512, Val351, Ala63, and Tyr62	Tyr501, tyr369, Val36, Tyr338, Trp335, Ala334, Ala332, Val329, Phe490	Val213, Ile227, Cys191, Leu41, Cys42, Cys58, Pro96, and Ile99	Tyr105, Phe34, Leu102, Ala99, Pro98, Pro71, and Ala72	Trp95, Val93, Ile92, Pro90, Leu40, Tyr78, Ala79	Met73, Phe135, Tyr133, Leu37, Tyr90, Met88, Phe96, Val61, and Leu63	Pro90, Val87, Tyr112, Cys115, Cys51, Ala50, and Trr42	Tyr79, Ala78, Tyr90, Met99, Met62, Met44, Leu37, and Ile30
Polar	Arg522, Glu411, His378, Asp358, Glu384, Lys368, Ser355, His353, Asn70, Asn66, and Glu143	Arg500, Arg381, Thr496, Glu389, His388, Asn494, Ser39, His365, Asp336, Glu362, His361, Ser333, His331, Lys346, and Asp43	Ser195, Ser190, Thr214, Ser215, Ser217, Arg218, Asp194, Glu60, Asp97, Hid57, Thr98, and Lys192	Gln36, Ser104, Ser101, Thr73, and Asp74	Lys94, Kys91, Gln89, Asn42, Lys41, and Glu44	Gln98, Lys99, Arg121, Asn101, and Asp102	Asn118, Gln116, Thr53, Ser48, Asn45, Glu52, and Glu69	Thr63, Ser64, Arg66, Thr75, Ser76, Lys118, Thr116, Thr128, and Lys130
Hydrogenous	Arg522, Glu411, Glu143, Asn70, Ala356, and Asn66	Asn494, Glu362, Asp336, Tyr338, Arg381, and Glu389	Ile227, Thr214, Gly216, Leu41, Cys42, Arg218, and Lys192	Ala72, Asp74, Tyr105, Gln36, Gly100, Gly103, Ser10, Gly100, and Ala99	Pro90, Lys91, Lys94, Glu77	Arg121, Glu33, Tyr133,Asp102, Gln98, and Asn101	Gly117, Thr53, and Thr42	Ala78, Tyr79, Thr128, Lys130, Arg66, Ser64, and Thr75

However, when considering the active site of the cystatin C receptor (PDB ID: 3GAX), another inflammatory marker, delphinidin-3-glucoside demonstrated a stronger interaction compared to other compounds, including tiliroside (Table 3). Van der Waals interactions were observed with residues Ala46, Cys83, Leu80, Leu27, Phe29, and Val31, while polar interactions occurred with residues Asn35, Asp28, His43, Arg45, Asn82, and Asp81. The hydrogen bonds formed by the compound involved residues Asn35 (2.83 Å), His43 (1.71 Å), Gly32 (3.24 Å), Asn82 (2.90 ), Asp81(2.32 Å), and Arg45 (2.09 and 3.24 Å) in the active site of the target (Figure 2I).

When compared to hydrochlorothiazide, as shown in Table 3, tiliroside exhibited significant interactions with α-1-microglobulin (PDB: 1B1K) and α-1-acid glycoprotein 1 (PDB: 3QKG), which are endogenous anti-inflammatory proteins that play a renal protective role. Tiliroside interacted with the active sites of the 1B1K target through its hydroxyl and carbonyl groups, forming four hydrogen bonds at distances of 1.96, 2.42, 2.68, and 1.92 Å (Figure 2G). Additionally, five hydrogen bonds were observed between α-1-acid glycoprotein target (PDB: 3QKG) and tiliroside, with bond distances ranging from 1.90 to 3.28 Å (Figure 2H).

**Drug likeness and ADMET prediction**

**Figure 3:**
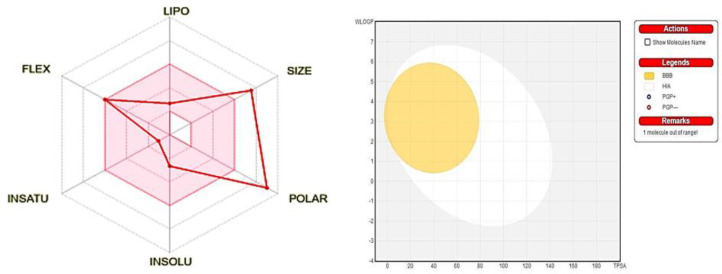
A) Bioavailability Radar drug likeness characteristic of Tiliroside**. **B) Schematic representation of perceptive evaluation of passive gastrointestinal absorption (HIA) and brain permeation (BBB) with Tiliroside in the WLOGP verses TPSA using BOILED Egg. BOILED‐Egg: Brain or IntestinaL EstimateD permeation predictive model; TPSA: topological polar surface area; WLOGP: Wildman and Crippen

The compounds were evaluated for their drug likeness and ADMET properties, including pharmacokinetic and toxicity profiles. Further analysis was conducted to assess compliance with Lipinski's Rule of Five, and most of the compounds met the criteria without any violations. However, tiliroside demonstrated moderate solubility with a calculated solubility value of -2.65 and a Log p value of -0.84. It had a molecular weight exceeding 500 Dalton and more than 5 hydrogen bond donors. However, it violated the Lipinski rule in terms of the number of H-acceptors, while the other compounds adhered to the rule (Figure 3A).

The pharmacokinetic and ADMET profiles of the compounds were examined to predict their toxicity parameters, and it was observed that tiliroside fulfilled the criteria for drug likeness. The compounds displayed good absorption and permeability, indicating moderate absorption. Key ADMET descriptors such as Human Intestinal Absorption (HIA) and blood-brain barrier (BBB) demonstrated favorable profiles. Additionally, tiliroside was not found to be an inhibitor of CYP450 enzymes (3A4, 2D6, and 2C9), which are involved in drug metabolism. Most of the compounds exhibited non-carcinogenic, non-hepatotoxic, and non-AMES toxic properties, aligning with the clinical and biological assays (Figure 3B).

## Discussion

This single-center, double-blind, randomized controlled clinical trial aimed to evaluate the efficacy of HS pills in treating hypertension in patients with CKD. Additionally, the study investigated the selective ACE inhibitory effects of HS compounds and their activation/inhibition properties on kidney targets through molecular docking. Both the control and HS groups showed a decrease in SBP and DBP among participants with CKD. Furthermore, the HS pill demonstrated a significant reduction in TG and cholesterol compared to the control group over the 90-day treatment period. Although a decrease in LDL-C was observed in the HS group, it did not reach statistical significance compared to the baseline. The molecular docking study revealed the beneficial effects of HS compounds, particularly anthocyanidins, in reducing blood pressure and targeting inflammation in CKD, consistent with the clinical findings.

Hypertension and CKD have a bidirectional relationship, contributing to the progression and severity of each other. Kidney dysfunction leads to increased sympathetic tone, endothelial dysfunction, and dysregulation of the renin-angiotensin-aldosterone system (RAAS), which amplifies hypertension (Ku et al., 2019 ▶). Conversely, hypertension causes oxidative stress and renal hypoxia, promoting the progression of CKD and hypertension (Pugh et al., 2019 ▶). Angiotensin II (Ang II) plays a role in inflammation and endothelial dysfunction by initiating an inflammatory cascade and reactive oxygen species (ROS) production (Dinh et al., 2014 ▶; Ku et al., 2019 ▶; Pugh et al., 2019 ▶; Xu et al., 2022 ▶), which contribute to inflammatory processes and CKD progression. Thus, hypertension and Ang II can activate oxidative and inflammatory pathways, leading to the progression of CKD or vice versa (Dinh et al., 2014 ▶). Importantly, the kidney is susceptible to the effects of hypertension due to the close interaction between them through the RAAS, which involves dysregulation of renin secretion and body fluid balance (Dong et al., 2021 ▶). Several anti-inflammatory and antioxidant proteins, such as alpha-1-microglobulin and α1-acid glycoprotein, as well as inflammatory markers including complement factor D, β-trace protein, β-2-microglobulin, retinol-binding protein 4, and cystatin-C, are implicated in both CKD and hypertension (Aleksenko et al., 2020 ▶; Argyropoulos et al., 2017 ▶; Farjo et al., 2012 ▶; McCullough et al., 2014 ▶; Okura et al., 2010 ▶; White, Ghazan-Shahi, and Adams, 2015 ▶). Additionally, there is a connection between oxidative stress/inflammation and selective nACE inhibition, which increases N-acetyl-SDKP levels and exerts anti-inflammatory and antifibrotic effects in the heart, kidneys, and vasculature (Xu et al., 2022 ▶).

Furthermore, HS has a long history of use in various medical fields, including its remarkable clinical effects as an anti-hypertensive, nephropathy, and diuretic agent. Phytochemical analysis of HS has identified potential secondary metabolites such as phenolic acids, flavonoids, anthocyanidins, organic acids, and phytosterols (Kamyab et al., 2022 ▶). Additionally, HS has demonstrated effectiveness as an anti-bacterial, anti-inflammatory, anti-diabetic, and antioxidant agent. The presence of phenolic compounds, particularly anthocyanidins, in HS is responsible for its antioxidant and anti-inflammatory effects (Al Snaf, 2018 ▶; Izquierdo-Vega et al., 2020 ▶; Pérez-Torres et al., 2013 ▶). Previous preclinical and clinical studies have indicated that HS administration provides beneficial effects, including anti-hypertensive effects, improvement in endothelial and renal function, diuretic properties, and anti-hyperlipidemic effects. These beneficial effects of HS are attributed to the antioxidant and anti-inflammatory properties of its active ingredients (Joven et al., 2014 ▶; Alarcón-Alonso et al., 2012 ▶; Nwachukwu et al., 2017 ▶; Nwachukwu et al., 2015 ▶; Hopkins et al., 2013 ▶; McKay et al., 2010 ▶).

Moreover, hypertension is a significant complication in patients with CKD, and its occurrence is mediated by ACE-2 and the RAAS pathway, with the kidneys playing a crucial role as the main regulator of blood pressure (Pugh et al., 2019 ▶). ACE inhibition and low levels of ACE-2 receptors lead to increased renal potassium excretion and sodium retention, resulting in electrolyte imbalances such as hypokalemia and hypernatremia (Beck et al., 2020 ▶). Interestingly, in this study, HS maintained electrolyte levels (Na^+^ and K^+^) within the baseline range, suggesting that HS compounds may directly or indirectly be effective in managing cardiac dysfunction in CKD through selective ACE inhibition and the regulation of ACE and electrolyte interactions. Additionally, the observed reduction in cholesterol and TG is a beneficial outcome in hypertensive patients, as elevated levels of these lipid components are risk factors for coronary heart disease.

However, the molecular docking results of our study suggest the potential of HS compounds, particularly tiliroside, to exhibit anti-inflammatory effects and provide renal and cardiac protection against elevated inflammation markers observed in both cardiac and kidney diseases. These markers include beta-2-microglobulin, β-Trace protein, cystatin C, retinol-binding protein 4, and complement factor D. Notably, the anthocyanidins and flavonoids present in HS may act as new inhibitors for these inflammatory markers, which are implicated in CKD and cardiovascular diseases (Aleksenko et al., 2020 ▶; Argyropoulos et al., 2017 ▶; Farjo et al., 2012 ▶; McCullough et al., 2014 ▶; Okura et al., 2010 ▶; White, Ghazan-Shahi, and Adams, 2015 ▶), thereby providing more effective protection against the inflammatory state in CKD. Additionally, HS compounds stimulate the production of endogenous anti-inflammatory and antioxidant reno-protective molecules, such as α-1-microglobulin and α-1-acid glycoprotein 1, enhancing their physiological anti-inflammatory and immunoregulatory functions (Kristiansson et al., 2020 ▶; Watanabe et al., 2021 ▶). Therefore, the rational interplay between these endogenous proteins and HS compounds, particularly tiliroside, suggests their potential as pharmacological agents for the therapeutic development of kidney and cardiac damage. However, to gain a deeper understanding of the precise effects of HS ingredients, further investigations into the interplay between these ingredients and protein targets in relation to renal function are required.

This study has several limitations that should be noted. Firstly, the small sample size of the study may have impacted the statistical power and generalizability of the findings. Another major limitation is the inability to investigate time-dependent changes in blood pressure and secondary biochemical outcomes, which could have provided important insights. Additionally, the lack of molecular studies on patients prevented the determination of changes in inflammatory markers during the observation period.

The present study suggests that HS pills may be an effective adjunct to valsartan in reducing blood pressure with improving lipid profile in CKD patients and could serve as a potential candidate to reduce side effects of hydrochlorothiazide. Significant improvements in both systolic and diastolic blood pressures were observed with HS pill treatment, accompanied by reductions in TG and cholesterol levels. Importantly, HS administration was found to be safe, supported by biological assays and molecular docking studies. Furthermore, our findings provide valuable therapeutic insights into the use of HS ingredients as safe pharmacological agents for enhanced protection in hypertensive CKD patients. However, given the limitations of this study, further clinical trials with larger sample sizes and more comprehensive assessments are warranted to validate these findings and provide more detailed information.

## Conflicts of interest

Mohammad Hadi Soleimani is the CEO of Giah Essence Phytopharmaceutical Co
